# Inhibitory Effects of *Morinda officinalis* Extract on Bone Loss in Ovariectomized Rats

**DOI:** 10.3390/molecules14062049

**Published:** 2009-06-08

**Authors:** Nan Li, Lu-Ping Qin, Ting Han, Yan-Bin Wu, Qiao-Yan Zhang, Hong Zhang

**Affiliations:** 1 Department of Pharmacognosy, School of Pharmacy, Second Military Medical University, Shanghai 200433, China; 2 Academy of Integrative Medicine, Fujian College of Traditional Chinese Medicine, Fujian 350108, China

**Keywords:** *Morinda officinalis*, ovariectomy, osteoporosis, serum biochemistry, bone mineral density

## Abstract

The present study was undertaken to investigate the protective effects of ethanol extract from the root of *Morinda Officinalis* (RMO) on ovariectomy-induced bone loss. Administration of RMO extract increased trabecular bone mineral content and bone mineral density of tibia, improved the levels of phosphorus (P), calcium (Ca) and OPG, decreased the levels of DPD/Cr, TRAP, ACTH and corticosterone, but did not reverse the levels of ALP, TNF-α and IL-6 in serum of ovariectomized rats. These findings demonstrated that RMO extract reduced bone loss in ovariectomized rats, probably via the inhibition of bone resorption, but was not involved with bone formation. Anthraquinones and polysaccharides from *Morinda officinals* could be responsible for their antiosteoporotic activity, and the action mechanism of these constituents needs to be further studied. Therefore, RMO has the potential to develop a clinically useful antiosteoporotic agent.

## Introduction

Osteoporosis, characterized by a loss of bone mass, is a major health problem, especially for elderly women. After the onset of menopause, a reduction in the circulating level of estrogen results in bone loss and increases the incidence of osteoporosis [1]. Classical hormone replacement therapy (HRT) has been recommended to prevent and treat postmenopausal osteoporosis for many years. However, in 2002, the American National Institute of Health stopped a clinical trial with HRT in healthy postmenopausal women due to the higher incidence of breast cancer, heart attack, and stroke and blood clots [2]. Traditional Chinese medicines have been applied to prevent and treat postmenopausal osteoporosis in clinical practice for thousands of years. And these medicines with fewer side effects are more suitable for long-term use compared with chemically synthesized medicines.

The root of *Morinda Officinalis* How (RMO), named “Ba-ji-tian”, has been used as a treatment for women’s diseases relative to estrus and as a stimulant of sex drive for its effects of nourishing kidney [3], which is related to its estrogen-like effects [4]. In south China, Hong Kong and Macao, this plant has been developed into various health foods, such as “Ba-ji-tian wine”, “Ba-ji-zi-bu Gao”. Pharmacological studies showed that RMO extract enhanced the expression of core-binding factor α1 (cbfα1), a key transcription factor for osteoblast differentiation [5], increased the proliferation, alkaline phosphatase (ALP) activity and osteocalcin of osteoblast [6]. Animal experiments also found that RMO aqueous extract could evidently suppress the bone loss in mice with osteoporosis induced by sciatic neurectomy surgery [7]. However, estrogen deficiency is the most important factor for postmenopausal osteoporosis. Therefore, it is essential to evaluate the RMO antiosteoporotic activity in ovariectomized animal model, and to elucidate the antiosteoporotic potential and possible action mechanism.

## Results

### Chemical constituents HPLC analysis

**Figure 1:**
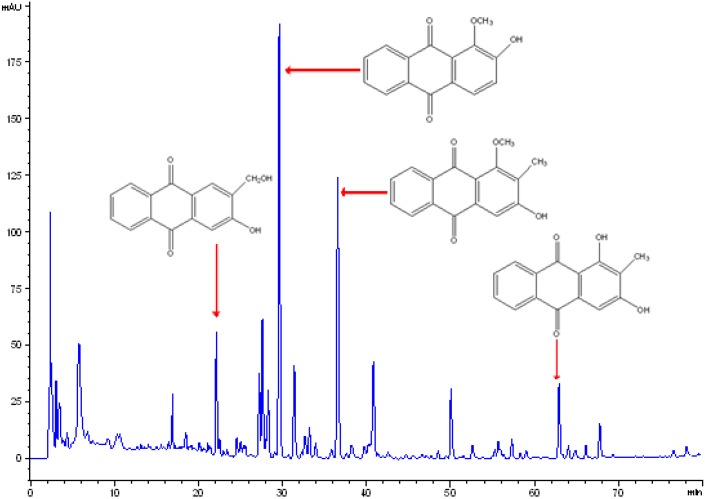
HPLC chromatogram of RMO. The major peaks were identified by comparison with standard compounds, and were respectively 2-hydroxymethyl-3-hydroxy anthraquinone, 2-hydroxy-1-methoxy anthraquinone, rubiadin-1-methyl ether and rubiadin.

The ethanolic extract of the roots of *M. officinalis* was further fractionated into petroleum ether, ethyl acetate, *n*-BuOH and H_2_O fractions. Among them, the ethyl acetate fraction dose-dependently stimulated osteoblast proliferation and ALP activity, and inhibited osteoclastic TRAP activity. Furthermore, activity-guided fractionation of the ethyl acetate fraction was carried out for the isolation of active constituents. Further fractionation and separation by several chromatographic methods yielded seven anthraquinones and one coumarin. These compounds were identified as physcion, rubiadin-1-methyl ether, 2-hydroxy-1-methoxyanthraquinone, 1,2-dihydroxy-3-methylanthraquinone, 1,3,8-trihydroxy-2-methoxyanthraquinone, 2-hydroxymethyl-3-hydroxyanthraquinone, 2-methoxy-anthraquinone and scopoletin, respectively, by comparison of their spectroscopic data (^1^H-, ^13^C-NMR and MS) with those reported in the literature [8]. The HPLC analysis indicated there are anthraquinones in the RMO extracts, and the major peaks were identified by comparison with standard compounds, and were respectively 2-hydroxymethyl-3-hydroxy anthraquinone, 2-hydroxy-1-methoxy anthraquinone, rubiadin-1-methyl ether and rubiadin (Figure 1).

### Body and uterine weight

Unlike the nylestriol, which inhibit the body and uterine weights gain of ovariectomized (OVX) rats, administration of the plant extract did not present any adverse effect on the treated animals as observed by the monitoring of their body and uterine weights. There was no significant difference between initial body weights of the six groups at the beginning of the study. At the end of the study, there was a significant weight gain of OVX and OVX + RMO animals. This resulted in a significant difference between OVX control and untreated control, as well as between OVX + RMO and untreated controls, whereas there was no difference between OVX and OVX + RMO animals. As expected, mean uterine weight of OVX animals was significantly lower than that of controls (Table 1).

**Table 1:** Comparison of body and uterine weight among groups (n=10)**.**

Groups	Body weights (g)		Uterine weight (g)
	Initial	Final	
Untreated	301±18	325±16	0.76±0.21
OVX control	312±25	401±41^∆∆∆^	0.28±0.24^∆∆∆^
Nylestriol	306±20	314±17^***^	0.49±0.16^*^
OVX+0.5g/kg RMO	325±41	433±62	0.16±0.029
OVX+1.0g/kg RMO	319±31	399±49	0.27±0.248
OVX+2.0g/kg RMO	311±23	399±55	0.25±0.200

All values are expressed as mean ± S.E.M. ^∆∆∆ ^*P* < 0.001 vs. untreated control group; ^*^P < 0.05, ^***^P < 0.001 vs. OVX group.

### Bone mineral content (BMC) and bone mineral density (BMD)

Peripheral quantitative computed tomography (*p*QCT) was used to evaluate the bone mineral content (BMC) and bone mineral density (BMD) of rats, which can separately examine cortical and trabecular bone changes. As shown in Table 2 and Table 3, 12 weeks after ovariectomy tibia BMC and BMD significantly decreased in total and trabecular bone compared with untreated rats, but did not change in cortical bone. Administration of RMO extract at dose of 2g/kg, total and trabecular bone BMC and BMD in tibia both significantly increased, and RMO at dose of 1g/kg also significantly increased the trabecular BMD in tibia compared with OVX control. These results indicated that RMO extract improved BMC and BMD of total and trabecular bone, decreased bone loss induced by ovariectomy.

**Table 2:** Effects of RMO on bone mineral content in OVX rats (n=10).

Groups	Total bone content (mg/mm)	Trabecular bone content (mg/mm)	Cortical bone content (mg/mm)
Untreated	14.2±1.4	3.4±0.5	8.4±0.9
OVX control	10.1±0.6^∆∆∆^	1.6±0.4^∆∆∆^	7.5±0.3
Nylestriol	12.3±0.4^*^	3.0±0.8^***^	7.6±0.4
OVX+0.5g/kg RMO	10.8±0.7	1.6±0.3	7.9±0.5
OVX+1.0g/kg RMO	11.4±0.9	2.0±0.6	8.0±0.4
OVX+2.0g/kg RMO	11.5±0.6	2.1±0.3^*^	8.2±0.8

All values are expressed as mean ± S.E.M., ^∆∆∆ ^*P* < 0.001 *vs*. untreated control group; ^*^P < 0.05, ^***^P < 0.001 *vs*. OVX group.

**Table 3:** Effects of RMO on bone mineral density (BMD) in OVX rats (n=10).

Groups	Total BMD (mg/cm^3^)	Trabecular BMD (mg/cm^3^)	Cortical BMD (mg/cm^3^)
Untreated	725±65	398±51	1125±31
OVX control	528±30^∆∆∆^	159±18^∆∆∆^	1117±58
Nylestriol	646±65^**^	332±58^***^	1109±36
OVX+0.5g/kg RMO	564±46	169±76	1130±18
OVX+1.0g/kg RMO	590±66	218±55^*^	1120±29
OVX+2.0g/kg RMO	591±33^*^	205±41^*^	1130±30

All values are expressed as mean ± S.E.M., ^∆∆∆ ^*P* < 0.001 vs. untreated control group; ^*^P < 0.05, ^***^P < 0.001 vs. OVX group.

### Serum P and Ca levels

Serum phosphorus and calcium levels indirectly reflect bone metabolism to some extent. As shown in Table 4, serum phosphorus and calcium levels significantly decreased in OVX control rats compared with untreated rats. Administration of the RMO extract at dose of 2g/kg and nylestriol strongly increased the serum P and Ca levels in OVX rats. These results indicated that RMO extract improved the P and Ca absorption, and bone matrix formation.

**Table 4:** Effects of RMO on serum P, Ca, DPD/Cr, TRAP and ALP levels in OVX rats (n=10).

Groups	P (mmol/L)	Ca (mmol/L)	DPD/Cr	TRAP (u/L)	ALP (u/L)
Untreated	2.35±0.21	2.83±0.09	0.24±0.08	9.5±0.8	50.6±15.9
OVX control	1.91±0.13^∆^	2.65±0.11^∆^	4.88±0.66^∆∆∆^	11.8±1.3^∆^	76.2±12.3^∆∆^
Nylestriol	2.30±0.31^*^	2.76±0.14^*^	0.49±0.12^**^	8.6±0.9^*^	53.8±14.1^*^
OVX+0.5g/kg RMO	1.82±0.31	2.58±0.34	0.17±0.09^**^	0.8±0.4^***^	73.4±28.0
OVX+1.0g/kg RMO	2.03±0.52	2.79±0.14	0.14±0.16^**^	0.6±0.4^***^	67.7±13.2
OVX+2.0g/kg RMO	2.41±0.34^**^	2.91±0.18^*^	0.29±0.38^**^	1.1±0.9^**^	74.0±23.0

All values are expressed as mean ± S.E.M. ^∆ ^*P* < 0.05, ^∆∆ ^*P* < 0.01, ^∆∆∆ ^*P* < 0.001 vs. untreated group; ^*^P < 0.05, ^**^P < 0.01, ^***^P < 0.001 vs. OVX group.

### Serum DPD/Cr, TRAP and ALP

Serum DPD/Cr and TRAP levels are biochemical markers of bone resorption, and ALP is one of bone formation markers. Ovariectomy induced high bone turnover in rats, serum DPD/Cr, TRAP and ALP levels also increased. Nylestriol decreased serum DPD/Cr, TRAP and ALP levels, and inhibited high bone turnover in OVX rats. Administration of RMO extract at the dose of 0.5-2g/kg significantly decreased serum DPD/Cr and TRAP levels, but did not reverse serum ALP levels (Table 4). These results showed that RMO extract strongly increased bone density by inhibiting bone resorption.

### Serum OPG, IL-6 and TNF-α

OPG (osteoprotegerin) is an endogenous protein produced by osteoblastic cells, which inhibits osteoclast formation, activation, and survival. IL-6 and TNF-α stimulate osteoclast to increase the bone resorption. Ovariectomy decreased serum OPG levels, increased serum TNF-α and IL-6 levels. Nylestriol efficiently increased serum OPG, and decreased serum TNF-α and IL-6 levels. RMO extract at the dose of 0.5-2g/kg also significantly increased OPG levels, but did not evidently decrease serum TNF-α and IL-6 levels in ovariectomized rats (Table 5).

**Table 5:** Effects of RMO on serum OPG, TNF-α, IL-6, ACTH and Corticosterone levels in OVX rats (n=10).

Groups	OPG (pg/mL)	TNF-α (pg/mL)	IL-6 (pg/mL)	ACTH (pg/mL)	Corticosterone (nmol/L)
Untreated	692±86	29.0.0±6.7	245±23	223±6	131±2
OVX control	553±77^∆^	38.9±11.1^∆^	345±29^∆^	258±7^∆∆∆^	138±2^∆∆∆^
Nylestriol	689±66*	24.9±10.6*	288±48*	230±10^***^	134±2^**^
OVX+0.5g/kg RMO	2127±335^***^	51.9±38.5	343±46	241±16^*^	135±2^**^
OVX+1g/kg RMO	1838±264^***^	53.7±38.0	337±39	227±12^***^	135±2^*^
OVX+2g/kg RMO	2217±559^***^	59.0±39.9	326±38	227±13^***^	134±1^***^

All values are expressed as mean ± S.E.M. ^∆ ^*P* < 0.05, ^∆∆∆ ^*P* < 0.001 vs. untreated group; ^*^P < 0. 05, ^**^P < 0.01, ^***^P < 0.001 vs. OVX group.

### Serum ACTH and corticosterone

Ovariectomy induced a marked rise in serum ACTH and corticosterone production in rats, compared with the untreated group. The treatment with RMO extract at the dose of 0.5-2g/kg and nylstriol significantly decreased ACTH and corticosterone to normal levels in ovariectomized rats. These results showed that RMO extract could modulate the pituitary-adrenal function and endocrine balance (Table 5).

## Discussion

Currently there exist two well-established small animal models of local osteoporosis: the rat ovariectomy (OVX) and the immobilization (IM)-induced bone loss models. The OVX rat is an excellent preclinical animal model that correctly emulates the important clinical feature of the estrogen depleted human skeleton and the response of therapeutic agents [9]. Rapid postmenopausal osteoporosis occurring in female rats following ovariectomy is characterized by a decrease in trabecular density and a deterioration of bone architecture, especially a decrease of the total number of trabeculae and an increase of the number of their perforations. The quantitative loss of bone and the changes of its internal structure are responsible for the increased fracture risk in postmenopausal osteopenia [1]. Although the effects of RMO on experimental osteoporosis in sciatic neurectomized mice, an IM-induced bone loss model, has been investigated [7], which could not across-the-board assess antiosteoporotic activity of RMO. In the present study, the antiosteoporotic effect of RMO ethanol extract was evaluated via determining bone mineral density and serum biochemical parameters. Following administration of RMO extract as nylestriol for 12 weeks, BMC and BMD of tibia total and trabecular bone significantly increased in ovariectomized rats. These results indicated that RMO ethanol extract had evidently antiosteoporotic effect on ovariectomized rats.

The use of dietary phyto-oestrogens as a possible option for the prevention of osteoporosis has raised considerable interest because of the increased concern about the risks associated with the use of hormone-replacement therapy [10]. Previous studies have reported that RMO has estrogen-like effects [4]. Thus, we inferred that the antiosteoporotic activity of RMO may be related with its estrogen-like activity. Increasing evidence indicates that not only uterine and bone tissue but also fat tissue is estrogen receptive [11,12]. Both receptor types, ERα and ERβ, are demonstrated in human lipocytes [13]. In our study, unlike nylestriol, which significantly decreased body weight and increased uterine weight in ovariectomized rats, RMO ethanol extract could not affect their body weight (indirectly reflection of fat tissue weight) and uterine weight. These results showed that RMO ethanol extract did not reveal estrogen-like activity on uterus and fat tissue in ovariectomized rats, and this lack of uterotrophic activity could be beneficial to reduce the risk of endometrial, breast or ovarian cancer associated with estrogen treatment [14,15,16]. Maybe it is the diversity of chemical composition that enables RMO extract to inhibit bone loss without side effects of estrogens, which could be counteracted by interaction of these chemical constituents.

DPD and tartrate-resistant acid phosphatase (TRAP), important markers of bone resorption, positively correlate with histomorphometric indice of bone resorption [17]. Serum alkaline phosphatase (ALP) activity is an indicator of osteoblast activity in bones and a marker of bone formation [18]. It is well-known that estrogen decreases serum levels of these markers [19,20]. In our study, serum levels of DPD/Cr, TRAP and ALP were reduced by Nylestriol in OVX rats, and RMO extract was effective only in reducing DPD/Cr and TRAP levels. It seemed that RMO extract, dissimilar to nylestriol, did not affect the bone formation, but evidently inhibited bone resorption in OVX rats. These results also occur in other investigation. It has been reported that the extracts of *Wedelia calendulacea Less.*, containing isoflavanoids, had a definite protective effect on bone loss in ovariectomized rats, inhibited the TRAP activity and did not inhibit the ALP activity. The reason for producing these differences between estrogen and medicinal plants containing phytoestrogen need to be further studied [21]. Ovariectomy induces high bone turnover in rats and both higher bone formation and lower bone resorption may be of benefit to inhibition of bone loss induced by Ovariectomy.

Factors influencing bone density may be local or systemic [22]. Sex hormones are generally considered systemic mediators of bone density, yet their effects on bone metabolism are exerted mainly via local mechanisms. Cytokine function is closely related to hormonal status. Osteoclast is a terminally differentiated multinucleate cell of hematopoietic origin with unique ability to resorb bones [23]. Estrogen, as well as cytokines, is important regulators of this osteoclast formation process [24]. Estrogen-dependent osteoclast cytokines include tumor necrosis factor-α (TNF-α) and interleukin-6 (IL-6). IL-6 and TNF-α induce bone resorption by acting both directly and indirectly on osteoclasts [25]. Our investigation demonstrated that the serum levels of IL-6 and TNF-α in ovariectomized rats were not modified by the administration of RMO extract, but reduced by nylestriol. These results showed that RMO extract inhibiting bone loss might be irrelative to IL-6 and TNF-α. However, it has been reported that polysaccharides from *Morinda officinalis* could reduce the level of IL-6 and TNF-α in ovariectomized rats [26], which is not consistent with our results. Because the ethanol extract of *Morinda officinalis* mainly contains anthraquinones, and barely polysaccharides, it could not produce the same bioactivity as polysaccharides.

Osteoprotegerin (OPG) is an endogenous protein of the tumor necrosis factor receptor superfamily produced by osteoblastic cells, which inhibits osteoclast formation, activation, and survival by interrupting the interaction between receptor activator of NF-kappa B ligand (RANKL) and its receptor, RANK, by binding to RANKL as a decoy receptor with higher affinity than RANK [27]. IL-1β and TNF-α have been reported to up-regulate the expression of OPG as well as RANKL in human osteoblast lineage cells, and IL-6 does not regulate them. Thus, the effect of these cytokines in stimulating osteoclastogenesis may depend on the ratio of OPG: RANKL generated in the bone microenvironment [28,29]. The present study showed that RMO extract increased the OPG level, but not TNF-α and IL-6. Therefore, the RMO extract may directly stimulate osteoblast to produce OPG, but not indirectly increase OPG level via an increase of TNF-α. The increased OPG level enhanced the ration of OPG: RANKL, and decreased the bone resorption. Our previous studies indicated that anthraquinones from RMO have an antiosteoporotic activity. These results also have been verified. Some anthraquinones have been reported to possess an antiosteoporotic activity. Diacerein, an anthraquinone, has been shown to inhibit osteoclastic bone destruction through the inhibition of RANKL expression and the increase of OPG expression in MC3T32 E1 cells [30]. Maybe, it is the anthraquinones in RMO that stimulate the production of OPG, inhibit osteoclastogenesis, and increase the trabecular BMC and BMD of ovariectomized rats.

The hypothalamo-pituitary-adrenal (HPA) axis is activated in many stress-induced neuroendocrine and metabolic responses with negative effects on the reproductive axis. Administration of adrenocorticotropin (ACTH) or glucocorticoids could inhibit luteinizing hormone release and ovulation in animals and women. On the other hand, the activity of the HPA axis is modulated by estrogen, as shown by many studies in female rats and humans. Ovariectomy reduced ACTH and corticosterone levels, which were restored to normal by administration of estrogen [31]. The responses of ACTH and corticosterone to stress in ovariectomized rats were less than in normal rats, and the same was found for the steroids responses [32,33]. In the present study, however, the serum levels of ACTH and corticosterone evidently increased in ovariectomized rats, which seemed incompatible with previous reports. In fact, ovariectomized rats were observed only from hours to 21 days in previous studies, but we determined the serum levels of ACTH and corticosterone in rats ovariectomized after 12 weeks. It is well-known that chronic and excessive administration of glucocorticoids could induce osteoporosis. The glucocorticoids antagonize gonadal function, inhibit the osteoanabolic action of sex hormones, reduce intestinal absorption of calcium, and further lead to a negative calcium balance [34]. Excessive glucocorticoid-induced direct impairment of osteoblast and osteoclast function results in the reduction of bone remodeling and the diminishment of repair of microdamage in bones. In our study, RMO extracts, as nylestriol, significantly decreased the serum levels of ACTH and corticosterone in rats ovariectomized after 12 weeks. These results illustrated that antiosteoporotic activity of RMO extract was relative to its estrogen-like activity and further inhibition of ACTH and corticosterone in ovariectomized rats.

The studies have found that many medicinal plants have the potential to prevent and treat osteoporosis. These plants could be divided into two categories: one is vitamin D-containing plants [35,36,37], and the other is phytoestrogen-containing plants [38,39]. Vitamin D_3_ has to be metabolized as a first step to 25(OH)D_3_ in the liver, which is further hydroxylated in the 1 position to form 1,25-dihydroxyvitamin D3 [1,25(OH)2D3]. 1,25(OH)_2_D_3_ is effective in improving bone mineral density and therefore suitable for the treatment of bone lesions, such as osteoporosis. Phytoestrogen refers to any plant substance or metabolite that induces biological responses in vertebrates and can mimic or modulate the actions of endogenous oestrogens usually by binding to oestrogen receptors. Three main classes have been identified: flavonoids, lignans, and coumestans. The common characteristic of these classes is that they are diphenolic compounds with structural similarities to natural and synthetic oestrogens and antioestrogens (an aromatic A ring with one hydroxyl group and a second hydroxyl group on the same plane of the A ring). Anthraquinones from *Morinda officinalis* have some structural similarities with phytoestrogens, and the ethanol extract also showed estrogen-like activity. Our previous studies found anthraquinones from *Morinda officinalis* increased osteoblast ALP activity and inhibited osteoclast TRAP activity and bone resorption. Maybe, these anthraquinones exert their antiosteoporotic activity by estrogen receptor pathway. In additional, polysaccharides from *Morinda officinalis* also reduced the bone loss in ovariectomized rats. The plant polysaccharides often have the modulatory effects on immune system. The *Morinda* polysaccharides inhibited the increase of TNF-α and IL-6 levels in ovariectomized rats. So *Morinda* polysaccharides regulate the bone metabolism by influence on the immune system of ovareictomized rats. As mentioned above, anthraquinones and polysaccharides all contributes to the antiosteoporosis of *Morinda officinalis*. The comprehensive effects of these chemical substances need to be further studied.

## Conclusions

In summary, the administration of RMO extract for 12 weeks significantly enhanced both BMC and BMD of tibia total and trabecular bone, the serum levels of P, Ca, OPG, and decreased the serum levels of DPD/Cr, TRAP, ACTH and corticosterone, and also did not affect the serum levels of ALP, TNF-α and IL-6 in ovariectomized rats. These results demonstrate significantly antiosteoporotic activity of RMO extract. However RMO extract, different from nylestriol, did not modify body and uterine weights, which was propitious to reduce the risk of endometrial, breast or ovarian cancer relative to estrogen treatment. The present study suggests that the antiosteoporotic effect of RMO extract is performed mainly via inhibition of bone resorption, but not involved with bone formation. Anthraquinones and polysaccharides from *Morinda officinals* could be responsible for their antiosteoporotic activity, and the action mechanism of these constituents needs to be further studied.

## Experimental

### Drugs and reagents

Nylestriol was purchased from Shanghai Hualian Pharmaceutical Co. Ltd. The reagent kits for measurement of calcium, inorganic phosphorus, tartrate-resistant acid phosphatase (TRAP), creatinine(Cr) and alkaline phosphatase (ALP) activity in serum were obtained from Fortune Bio-medical Engineering Co. Ltd. (Shanghai, P.R. China). ELISA kits for measurement of Serum DPD (ER055), IL-6 (3R035), TNF-α (3R080), ACTH (3B956), corticosterone (HR083) and OPG (2B019) were purchased from Rapid Bio^TM^, USA.

### Preparation of RMO extract

The crude RMO drug was purchased from Hua Yu TM Drug Corporation Ltd. (Shanghai, P.R. China) and identified by Professor Lu-Ping Qin of the Department of Pharmacognosy, School of Pharmacy of the Second Military Medical University, Shanghai. The voucher specimen is available in the herbarium of this Department. An amount of 1.5 kg of RMO was dried, pulverized and extracted with 75% ethanol using a reflux apparatus (Vacuum Pump, SHB-ⅢA, Shanghai Yukang Instrument Company; Rotary Evaporator, Shanghai Senco Technology Co. Ltd). The ethanol extract was concentrated under reduced pressure at 50°C, and then dissolved in an appropriate volume of distilled water. The concentration of the ethanol extract was adjusted to 0.05 g/mL, 0.1 g/mL, and 0.2 g/mL equivalent to crude drug for *in vivo* evaluation. 

### HPLC fingerprint analysis

The HPLC fingerprint analysis was performed on an Agilent 1200 series HPLC system including a quaternary pump, vacuum degasser, thermostatic column compartment and a diode array detector (DAD). An Agilent Extend–C18 column (4.6 ´ 250 mm, 5 μm) with an Extend C-18 guard column (4.6 ´ 10 mm, 5 μm) was used. Gradient elution was employed using solvent systems A (0.2% phosphoric acid) and B (acetonitrile) at ambient temperature. The gradient program used was as follows: initial 0–15min, A–B (95:5, v/v); 15–35min, linear change to A–B (70:30, v/v); 35–80min, linear change to A–B (60:40,v/v). The flow rate was 1.0 mL min^-1^ and column temperature was maintained at 30°C. The detector wavelength was set at 277 nm and an aliquot of 20 µL solution was injected for acquiring chromatograms. The major peaks identified by comparison with standard compounds [8] were respectively 2-hydroxymethyl-3-hydroxy anthraquinone, 2-hydroxy-1-methoxy anthraquinone, rubiadin-1-methyl ether and rubiadin (Figure 1).

### Animals and experimental protocol

Sixty female Sprague-Dawley rats of 4.5 months of age were purchased from SLACOM experimental animal company (Shanghai, P.R. China) and acclimated to conditions for 1 week before the experiment. The experimental animals were housed in an air-conditioned room with 12h/12h light-dark illumination cycles at constant temperature 24±0.5 °C and humidity (45-50%). Food and drinking water were supplied *ad labium*. The rats were weighed every week during the experiments. The 10 rats were mock-operated and treated with vehicle (deionized water) as aging control (untreated+Veh). The remaining rats were bilaterally ovariectomized and randomly divided into five groups with 10 per group. They were treated with vehicle (water), nylestriol (1 mg・kg-1, ig, weekly) or RMO extracts (0.5, 1.0 and 2.0 g・kg^-1^・d^-1^, ig) for 12 weeks. Rats received treatments starting from one day after surgeries. Success of ovariectomy was confirmed at necropsy by failure to detect ovarian tissue and by observation of marked atrophy of uterine horns. At the end of the treatment, the blood samples from all the groups were withdrawn by abdomen artery method to assess biochemical parameters. The uteri were removed and immediately weighed. This experiment was approved by the Bioethic Committee of the Second Military Medical University, and the procedures of the experiment were strictly according to generally accepted international rules and regulations.

### Bone mineral density (BMD) assay

The tibia was cleaned off adhering soft tissues, kept in 75% ethanol for a week to remain the constant BMD. The bone mineral density (BMD) and bone mineral content (BMC) were measured at 3 mm from the proximal epiphysis of right tibia with a peripheral quantitative computed tomography (*p*QCT) densitometry (Stratec TM XCT Research SA, Germany).

### Serum biochemical parameters assay

Serum calcium (Ca), inorganic phosphorus (Pi) concentration and serum alkaline phosphates (ALP), tartrate-resistant acid phosphatase (TRAP) and Serum creatinine (Cr) were measured on an automatic analyzer (Ciba-Corning 550, USA) using diagnostic reagent kit *in vitro* determination. Serum ACTH, corticosterone, IL-6, TNF-α, OPG and DPD were estimated using an Elisa kits according to product instructions. The linear range of the analytes is listed as follows and the samples were diluted up to 10 times to measure ACTH and corticosterone before measurement. ACTH (1-200 pg/mL), corticosterone (0.7-112 nmol/L), IL-6 (8-1000 pg/mL), TNF-α (8-1000 pg/mL), OPG (50-3000 pg/mL), DPD (0.5-100 nmol/L), ALP (0-700 u/L), TRAP (0.3-15 u/L), Ca (0.5-50 mmol/L), P (0.03-10 mmol/L).

### Statistical evaluation

Data were expressed as means ± S.E.M. Statistical analyses were carried out using a one-way analysis of variance (ANOVA) followed by Dunnett’s Multiple Comparison test (Chicago). Differences between the groups were considered statistically signiﬁcant at the P < 0.05 level, the level of significance set at * *P* < 0.05, ** *P* < 0.01 and *** *P* < 0.001.

## References

[1] Jee W.S.S., Yao W. (2001). Overview: animal models of osteopenia and osteoporosis. J. Musculoskel. Neuronal Interact..

[2] Rossouw J.E., Anderson G.L., Prentice R.L. (2002). Risks and benefits of estrogen plus progestin in healthy postmenopausal women: principal results from the women’s health initiative randomized controlled trial. J. Am. Med. Assoc..

[3] National Pharmacopoeia Commission of P.R. China (2005). Pharmacopoeia of the People’s Republic of China.

[4] Zhang Z.Q., Li Y., Yang M., Luo Z.P., Zhao Y.M. (2002). The effect of *Morinda officinalis* How, a Chinese traditional medicinal plant, on the DRL 72-s schedule in rats and the forced swimming test in mice. Pharmacol. Biochemi. Behavi..

[5] Wang H.M., Wang L., Li N. (2004). Effect of *Morinda Officinalis* on the expression of cbfα1 during the differentiation from BMSCs to the osteoblast. Chine. J. Trad. Med..

[6] Li N., Wang H.M., Lin X., Zheng L.P., Shen L., Wang L., Chen B.Y. (2004). Experimental study on effects of Radix *Morinda Officinalis* on biological specialty of osteoblast. Zhong Guo Yi Yao Xue Bao.

[7] Seo B., Ku S.K., Cha E.M., Park J.H., Kim J.D., Choi H.Y., Lee H.S. (2005). Effect of *Mornidae* Radix extracts on experimental esteoporosis in sciatic neurectomized mice. Phytother. Res..

[8] Wu Y.B., Zheng C.J., Qin L.P., Sun L.N., Han T., Jiao L., Zhang Q.Y., Wu J.Z. (2009). Antiosteoporotic activity of anthraquinones from *Morinda officinalis* on osteoblast and osteoclasts. Molecules.

[9] Kimmel D.B., Marcus R., Feldman D., Kelsey J. (1996). Animal models for *in vivo* experimentation in osteoporosis research. Osteoporosis.

[10] Branca F. (2003). Dietary phyto-oestrogens and bone health. Proc. Nutr. Soc..

[11] Ohlsson C., Hellberg N., Parini P. (2000). Obesity and disturbed lipoprotein profile in estrogen receptor-alpha-deficient male mice. Biochem. Biophys. Res. Commun..

[12] Anwar A., McTernan P.G., Anderson L.A. (2001). Site-specific regulation of oestrogen receptor-alpha and -beta by oestradiol in human adipose tissue. Diabetes Obes. Metab..

[13] Pedersen S.B., Bruun J.M., Hube F., Kristensen K., Hauner H., Richels B. (2001). Demonstration of estrogen receptor subtypes alpha and beta in human adipose tissue: Influences of adipose cell differentiation and fat depot localization. Mol. Cell Endocrinol..

[14] Mokbel K. (2003). Risk-reducing strategies for breast cancer--a review of recent literature. Int. J. Fertil Womens Med..

[15] Smith R.E. (2003). A review of selective estrogen receptor modulators and national surgical adjuvant breast and bowel project clinical trials. Semin. Oncol..

[16] Beck V., Rohr U., Jungbauer A. (2005). Phytoestrogens derived from red clover: an alternative to estrogen replacement therapy. J. Steroid Biochem. Mol. Biol..

[17] Kalu D.N. (1991). The ovariectomized rat model of postmenopausal bone loss. Bone Miner..

[18] Yilmaz B., Seyran A.D., Sandal S., Aydin M., Colakoglu N., Kocer M., Carpenter D.O. (2006). Modulatory effects of Aroclors 1221 and 1254 on bone turnover and vertebral histology in intact and ovariectomized rats. Toxicol. Lett..

[19] Erben R.G., Brunner K.S., Breig B. (2004). Long-term sensitivity of uterus and hypothalamus/pituitary axis to 17-beta-estradiol is higher than that of bone in rats. J. Bone Miner Res..

[20] Das A.S., Das D., Mukherjee M., Mukherjee S., Mitra C. (2005). Phytoestrogenic effects of black tea extract (Camellia sinensis) in an oophorectomized rat (Rattus norvegicus) model of osteoporosis. Life Sci..

[21] Annie S., Prabhua R.G., Malini S. (2006). Activity of Wedelia calendulacea Less. In post-menopausal osteoporosis. Phytomedicine.

[22] Raisz L.G. (1988). Local and systemic factors in the pathogenesis of osteoporosis. N. Eng. J. Med..

[23] Teitelbaum S.L. (2000). Bone resorption by osteoclasts. Science.

[24] Jilka R.L., Hangoc G., Girasole G., Passeri G., Williams D.C., Abrams J.S., Boyce B., Broxmeyer H., Manolagas S.C. (1992). Increased osteoclast development after estrogen loss: mediation by interleukin-6. Science.

[25] Kobayashi K., Takahashi N., Jimi E., Udagawa N., Takami M., Kotake S. (2000). Tumor necrosis factor alpha stimulates osteoclast differentiation by a mechanism independent of the ODF/RANKL-RANK interaction. J. Exp. Med..

[26] Zhu M.Y., Wang C.J., Zhang H.S., Pei X.W., Fen J.M. (2008). Protective effect of polysaccharides from *morinda officinalis* on bone loss in ovariectomized rats. Int. J. Biol. Macromol..

[27] Yasuda H., Shima N., Nakagawa N., Yamaguchi K., Kinosaki M., Mochizuki S., Tomoyasu A., Yano K., Goto M., Murakami A., Tsuda E., Morinaga T., Higashio K., Udagawa N., Takahashi N., Suda T. (1998). Osteoclast differentiation factor is a ligand for osteoprotegerin/osteoclastogenesis inhibitory factor and is identical to TRANCE/RANKL. Proc. Natl. Acad. Sci. USA.

[28] Hofbauer L.C., Dunstan C.R., Spelsberg T.C., Riggs B.L., Khosla S. (1998). Osteoprotegerin production by human osteoblast lineage cells is stimulated by vitamin D, bone morphogenetic protein-2, and cytokines. Biochem. Biophys. Res. Commun..

[29] Hofbauer L.C., Lacey D.L., Dunstan C.R., Spelsberg T.C., Riggs B.L., Khosla S. (1999). Interleukin-1β and tumor necrosis factor-α, but not interleukin-6 stimulate osteoprotegerin ligand gene expression in human osteoblastic cells. Bone.

[30] Wang L., Mao Y.J., Wang W.J. (2006). Inhibitory effect of diacere in on osteoclastic bone destruction and its possible mechan ism of action. Acta Pharm. Sin..

[31] Niyomchai T., Russo S.J., Festa E.D., Akhavan A., Jenab S., Vanya Q.J.T. (2005). Progesterone inhibits behavioral responses and estrogen increases corticosterone levels after acute cocaine administration. Pharmacol. Biochem. Behav..

[32] Burgess L.H., Handa R.J. (1992). Chronic estrogen-induced alterations in adrenocorticotropin and corticosterone secretion and glucocorticoid receptor-mediated functions in female Rats. Endocrinol..

[33] Fitch R.H., McGivern R.F., Redei E., Schrott L.M., Cowell P.E., Denenberg V.H. (1992). Neonatal ovariectomy and pituitary–adrenal responsiveness in the adult rat. Acta Endocrinol..

[34] Kitay J.I. (1963). Effects of oestradiol on pituitary–adrenal function in male and female rats. Endocrinology.

[35] Rosenberg S.V., Wehr U., Bachmann H. (2007). Effect of vitamin D-containing plant extracts on osteoporotic bone. J. Steroid Biochem. Mol. Biol..

[36] Boland R., Skliar M., Curino A., Milanesi L. (2003). Vitamin D compounds in plants. Plant Sci..

[37] Weissenberg M., Maoz A., Levy A., Wasserman R.H. (1988). Radioimmunoassay for rapid estimation of vitamin D derivatives in calcinogenic plants. Planta Med..

[38] Annie S., Prabhu R.G., Malini S. (2006). Activity of *Wedelia calendulacea* Less. in post-menopausal osteoporosis. Phytomedicine.

[39] Branca F. (2003). Dietary phyto-oestrogens and bone health. Proc. Nutr. Soc..

